# Exploring the influence of a financial incentive scheme on early mobilization and rehabilitation in ICU patients: an interrupted time-series analysis

**DOI:** 10.1186/s12913-024-10763-0

**Published:** 2024-02-24

**Authors:** Yoko Unoki, Sachiko Ono, Yusuke Sasabuchi, Yohei Hashimoto, Hideo Yasunaga, Isao Yokota

**Affiliations:** 1 Department of Biostatistics, Graduate School of Medicine, Hokkaido University, N15W7, Kita-ku, Sapporo, Hokkaido 0608638 Japan; 2 Department of Eat-loss Medicine, Graduate School of Medicine, The University of Tokyo, 7-3-1, Hongo, Bunkyo-Ku, Tokyo, 1130033 Japan; 3 Department of Real-world Evidence, Graduate School of Medicine, The University of Tokyo, 7-3-1, Hongo, Bunkyo-Ku, Tokyo, 1130033 Japan; 4 Department of Ophthalmology, Graduate School of Medicine, The University of Tokyo, 7-3-1, Hongo, Bunkyo-Ku, Tokyo, 1130033 Japan; 5 Department of Clinical Epidemiology and Health Economics, School of Public Health, The University of Tokyo, 7-3-1, Hongo, Bunkyo-Ku, Tokyo, 1130033 Japan

**Keywords:** Activities of daily living, Early mobilization, Early rehabilitation, Health policy, Interrupted time series analysis

## Abstract

**Background:**

Clinical guidelines recommend early mobilization and rehabilitation (EMR) for patients who are critically ill. However, various barriers impede its implementation in real-world clinical settings. In 2018, the Japanese universal healthcare coverage system announced a unique financial incentive scheme to facilitate EMR for patients in intensive care units (ICU). This study evaluated whether such an incentive improved patients’ activities of daily living (ADL) and reduced their hospital length of stay (LOS).

**Methods:**

Using the national inpatient database in Japan, we identified patients admitted to the ICU, who stayed over 48 hours between April 2017 and March 2019. The financial incentive required medical institutions to form a multidisciplinary team approach for EMR, development and periodic review of the standardized rehabilitation protocol, starting rehabilitation within 2 days of ICU admission. The incentive amounted to 34.6 United States Dollars per patient per day with limit 14 days, structured as a per diem payment. Hospitals were not mandated to provide detailed information on individual rehabilitation for government, and the insurer made payments directly to the hospitals based on their claims. Exposure was the introduction of the financial incentive defined as the first day of claim by each hospital. We conducted an interrupted time-series analysis to assess the impact of the financial incentive scheme. Multivariable radon-effects regression and Tobit regression analysis were performed with random intercept for the hospital of admission.

**Results:**

A total of 33,568 patients were deemed eligible. We confirmed that the basic assumption of ITS was fulfilled. The financial incentive was associated with an improvement in the Barthel index at discharge (0.44 points change in trend per month; 95% confidence interval = 0.20–0.68) and shorter hospital LOS (− 0.66 days change in trend per month; 95% confidence interval = − 0.88 – -0.44). The sensitivity and subgroup analyses showed consistent results.

**Conclusions:**

The study suggests a potential association between the financial incentive for EMR in ICU patients and improved outcomes. This incentive scheme may provide a unique solution to EMR barrier in practice, however, caution is warranted in interpreting these findings due to recent changes in ICU care practices.

**Supplementary Information:**

The online version contains supplementary material available at 10.1186/s12913-024-10763-0.

## Background

Early mobilization and rehabilitation (EMR) for patients who are critically ill has been indicated positive outcomes in critical care areas. EMR has been reported to improve activities of daily living (ADL) on the Barthel Index (BI) score at the time of hospital discharge [1, 2] and muscle strength on the Medical Research Council score [2–4]. Recent meta-analyses have reported that EMR decreased the incidence of intensive care unit (ICU)-acquired weakness [5, 6]. Other studies have suggested that EMR shortens ICU and hospital length of stay (LOS) [2, 7]. Likewise, the clinical practice guidelines emphasize EMR as a potential way to mitigate ICU-acquired weakness and impaired physical functions [8, 9].

Despite these findings and recommendations, several reports have indicated many barriers to EMR implementation in practice [10, 11]. A review classified barriers to EMR into patient-related, structural, ICU cultural, and process-related barriers [12]. EMR practice was associated with multidisciplinary rounds, setting goals for patients, the presence of dedicated physiotherapists, high-intensity physician staffing, and higher nurse staffing levels [13, 14].

Financial incentive schemes are used to motivate healthcare providers to improve the quality of care and patient health outcomes [15]. In April 2018, the Japanese universal health insurance system introduced a financial incentive scheme to provide EMR in the ICUs to overcome barriers and promote EMR as part of routine clinical care. An evaluation of this incentive may provide a unique solution to EMR barriers. However, the efficacy of this incentive on patient outcomes has only been reported in a small single-center study written in Japanese [16], and its nationwide effect remains unclear.

To address this knowledge gap, we examined the effectiveness of the financial incentive scheme for EMR on patient outcomes, using a national inpatient database in Japan.

## Methods

### Data source

Patient-level data were obtained from the Diagnosis Procedure Combination (DPC) database for this study. The database covers more than 7–8 million inpatients per year, which accounts for approximately 50% of all acute care hospitalizations in Japan [17]. The database consisted of administrative claims and discharge summary from approximately 1000 hospitals [18]. It includes the following information: hospital identification number, age, sex, admission date, ambulance use, discharge status (discharged to home, or other facilities, or in-hospital death), main diagnosis, pre-existing comorbidities, and post-admission complications coded with the International Classification of Diseases 10th Revision (ICD-10). ADL scores (BI) [19] at admission and discharge, medical procedures, and daily records of drug administration and devices were also used.

We also used hospital-level data were extracted from the Annual Report for Functions of Medical Institutions [20], and the Japanese Society of Intensive Care Medicine website [21]. The Annual Report for Functions of Medical Institutions as hospital structural information included the type of hospital, total inpatient days over 365 days in the ICU, and the number of full-time registered nurses and dedicated physiotherapists/occupational therapists/speech therapists in the ICU. Of these, we calculated the average patient-to-nurse ratio per shift in the ICU with the following equation: patient-to-nurse ratio per shift = total inpatient days/(the number of full-time registered nurses in the ICU*1800 h/24 h) [22, 23]. The total inpatient days indicated the sum of inpatient days in the ICU per year, and 1800 h represented the working hours per nurse per year. Lower patient-to-nurse ratios per shift meant better nurse staffing. Additionally, we used data pertaining to institutions certified as training facilities for intensivists authorized by the Japanese Society of Intensive Care Medicine. The designation of a training facility signifies its employment of at least one intensivist certified by the society on a full-time basis. The hospital-level data were integrated with patient-level data using the identical ward information code as a key. This study was approved by the Institutional Review Board of The University of Tokyo. Informed consent was waived because of the anonymous nature of the data.

### Patient selection

We included all patients admitted to the ICU between April 1, 2017 and March 31, 2019. Of these, we selected patients who stayed in the ICU for over 48 hours. ICU in the Japanese universal healthcare coverage system was defined as a 24-hour critical care unit with facilities necessary for treating patients who were critically ill, with at least one full-time physician on duty 24 hours a day and a nurse-to-patient ratio of more than 1:2. Patients who were younger than 15 years, were pregnant, and those who died during hospitalization were excluded because their ADL scores were not required to be reported. On account of data quality consideration, we excluded patients admitted to facilities with fewer than 10 overall eligible patients during the study period. In addition, hospitals not linked with the hospital structural information in the Annual Report for Functions of Medical Institutions were excluded.

### Study variables

Based on the literature search of known risk factors and clinical knowledge in the field, the study variables routinely collected were chosen from the Data sources (see additional file: Fig. S1) [13, 24–39]. We retrieved data on the patients’ age, sex, body mass index, smoking status (non-smoker or current/past smoker), type of admission (planned or urgent), and ambulance use. We identified the following procedures within 2 days of ICU admission: use of vasopressors (catecholamine and vasopressin), corticosteroids, neuromuscular blocking agents, aminoglycosides, blood transfusion, renal replacement therapy, mechanical circulatory support (such as intra-aortic balloon pump and extracorporeal membrane oxygenation), and mechanical ventilation. The ADL scores is required to assessed by a healthcare provider at admission and discharge and to be registered in the discharge summary. ADL score is scored on 0–3 scale for the following 10 categories, which can be multiplied by 5 to convert them into the BI; personal hygiene, bathing self, feeding, toilet, stair climbing, dressing, bowl control, bladder control, ambulation, chair/bed transfer [19]. Regarding the BI scores, a score of 100 suggested that the patient was independent of assistance from others, while a score closer to 0 indicated severe dependence and < 60 indicated severe functional dependence [40]. Thus, we categorized the BI scores into 0–55 (total and severe dependence), 60–96 (moderate and slight dependence), and 100 (independent). Comorbidities were converted into the Charlson comorbidity index developed by Quan et al. [41]. The Charlson comorbidity index was calculated using a routinely originated discharge summary defined by the ICD-10 codes, which did not change nationally in the report system within the study period. We used the Angus organ failure score to identify the severity of the illness, which consisted of the ICD-10 codes and Japanese medical procedure codes [42]. Higher scores indicated greater organ failure, and the maximum score was six (see additional file: Table S1). Primary diagnoses were classified into seven categories based on the ICD-10 codes.

The hospital characteristics investigated were hospital volume, type (academic or non-academic), patient-to-nurse ratio per shift in ICU, intensivists, and dedicated therapists. Hospital volume of patients was defined as the average annual number of study patients in each hospital.

### Outcomes and exposures

The primary and secondary outcomes were the BI at hospital discharge and hospital LOS, respectively. For the primary outcome, we used to absolute BI gain with the aim of correcting for the effect of the score at hospital admission. Mathematically, the formula is as follows: BI at hospital discharge - BI at hospital admission [43].

The exposure was the introduction of the financial incentive for EMR for critical patients defined as the first day of claim by hospitals. We highlighted the introduction date of the financial scheme for each hospital. Its requirements were: (1) a multidisciplinary team approach for early rehabilitation, which consisted of a full-time physician experienced in managing critical patients for over 5 years, a full-time certified nurse or certified nurse specialist in critical care nursing, and a full-time physiotherapist or occupational therapist with over 5 years of experience; (2) development and periodic review of the standardized rehabilitation protocol according to the expert consensus published by the Japanese society of intensive care medicine in January 2017 [44]; and (3) started rehabilitation within 2 days from ICU admission. The government paid hospitals 34.6 United States Dollars (USD) per day (1 USD = 144.52 Japanese yen in 2023) for each patient provided with a maximum of 14 days. The financial incentives were based on self-certification methods at the hospital level. Certification required the submission of documentation with information on the constituents enrolled in the multidisciplinary team (name, job title, years of experience, and documentary evidence), and information on the status of the standardized rehabilitation protocol (already developed or not) and frequency of review of the rehabilitation protocols. This financial incentives were not based on a performance-related compensation system linked to changes in outcomes. Thus, each hospital was not required to submit any process data or audits of the adherence or fidelity of EMR. At the patient level, information on rehabilitation efforts and intervention time was required to be logged in the medical record. Meanwhile, at the system level, hospitals received reimbursement for rehabilitation through the submission of billing codes to the Examination and payment agency, following the Japanese procedure codes. In the Japanese universal healthcare coverage system, patients typically cover a portion of their medical costs, while the remaining costs are reimbursed through insurance. The reimbursement for medical services is directly allocated to hospitals. The system functions based on either a fee-for-service or per diem payment structure.

### Statistical analysis

We specifically targeted patients admitted to hospitals that introduced the incentive within the study period in the main analysis. We defined the introduction of incentives as the first day of the incentive claim, set at time origin. We calculated the number of missing and removed all cases with missing data in any covariates except for the BI at admission and improvement at discharge. Patient characteristics were compared before and after the introduction of the financial incentive for EMR during ICU stay. Continuous and categorical variables were presented as means and standard deviations, and counts as proportions, respectively. Standardized differences were calculated to evaluate differences in baseline characteristics., with a difference within the range of − 0.2 to 0.2 considered negligible [45].

Interrupted time-series analyses were performed to assess the hypothesis that the introduction of the financial incentive payment for EMR was associated with improvements in the ADL scores at discharge and reduction in hospital LOS. The interrupted time-series analysis is a quasi-experimental design to evaluate the effectiveness of population-level health interventions [46]. We segmented the study period with the introduction of this financial incentive claims for each hospital. We hypothesized a level change and slope change in the outcomes occurring with no lag. This assumption was based on the similarity of the ICU facility criteria regarding staffing set by the Ministry of Health, Labour and Welfare and the composition of multidisciplinary team for early rehabilitation. Additionally, considering that more than a year had passed since the publication of the expert consensus [44], we anticipated the immediate functionality of the expert team. We also assumed an improvement in rehabilitation intervention rates over time. Furthermore, given that more than a year had passed since the publication of the expert consensus, it was assumed that the expert team would function immediately. We also assumed that rehabilitation intervention rates would increase gradually over time. Level changes indicated that the exposure accompanied the step of the outcome change immediately, whereas slope changes indicated that the exposure induced the gradual outcome change in the gradient of the trend [46]. The slope changes were represented in months, which referred to the effect size per month. An increase in slope and level change in BI at discharge indicates an ADL improvement at discharge, and a decrease in slope and level in LOS implies a shorter LOS.

To assess ADL improvement at discharge, multivariable random-effects regression analysis was performed with fixed slope, and random intercept for hospital of admission using the identical hospital code to account for clustering by hospital [47]. Additionally, we used a random-effect Tobit regression model to address right-censored hospital LOS with a random intercept for hospitals.

The Japanese universal healthcare coverage system established 90 days as the upper limit to claim acute hospital admission charges by the Ministry of Health, Labour and Welfare; we thus set 90 days as right censoring. The pre-and post-introduction periods, the time elapsed since the introduction for each hospital, and their interaction term were included in the analysis. The outcomes were adjusted for age, sex, body mass index, smoking status, Charlson comorbidity index, Angus organ score, BI at admission, emergency admission, ambulance use, days from hospitalization to ICU admission, drugs, procedures within 2 days, hospital volume, patient-to-nurse ratio, intensivist certified hospital, and dedicated therapist. We show the mean difference with confidence intervals (CIs) constructed using robust standard error. Statistical analyses were performed with R version 3.6.1 (R Foundation for Statistical Computing, Vienna, Austria) and STATA/MP, version 16.0 (StataCorp). All CIs were set to 95%.

#### Sensitivity analysis

We conducted several additional sensitivity analyses to further examine the stability of the results. First, we conducted the same analysis as the main analysis with the entire study population, including patients admitted to hospitals that either introduced or not the incentive within the study period. We set the issue of the financial incentive scheme on April 1, 2018, as the time origin. Second, to evaluate seasonality, we conducted the analyses at several intervals (see additional file: Fig. S2). Third, we fitted the mixed effect model using multiple imputations by chained equations. Additional analyses details are provided in the Methods sections of the Supplementary file.

#### Subgroup analysis

We conducted subgroup analyses stratified by age, type of admission, BI at admission, Angus organ failure score, and primary diagnosis based on the ICD-10 codes.

## Results

**Fig. 1:**
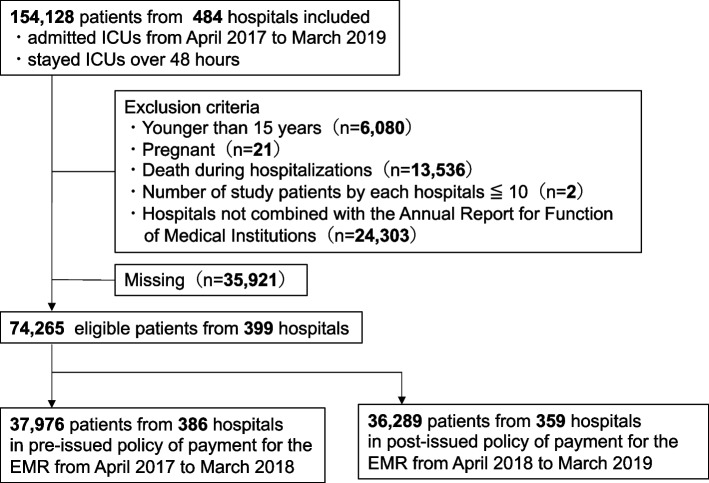
Flow chart of the patients

The cohort consisted of 45,836 patients from 169 hospitals during the two-year study period (Fig. 1). Of them, 26,033 and 19,803 were in the pre- and post-issue periods, respectively. Because the time origin of the post-introduction for financial incentives differed by hospitals, the number of patients in post-introduction was less than that in pre-introduction. Table 1 summarizes the characteristics of the patients. Patient characteristics were similar in that all covariate standardized differences were within ±(0.2)

**Table 1:** Patient characteristics

	Overall	Pre-introduction period	Post-Introduction period	
	(168 hospitals)	(164 hospitals)	(167 hospitals)	Std. Diff.
	(*n* = 33,568)	(*n* = 19,065)	(*n* = 14,503)	
Age, mean (SD)	70.42 (13.57)	70.26 (13.62)	70.n(13.51)	− 0.03
Male sex	21,174 (63.1)	12,057 (63.2)	9117 (62.9)	0.01
Body mass index (kg/m^2^), mean (SD)	22.89 (5.81)	22.84 (4.56)	22.96 (7.13)	−0.02
Smoking Status				
Current / Past smoker	17,963 (53.5)	10,260 (53.8)	7703 (53.1)	0.01
Charlson Comorbidity Index				
0	10,109 (30.1)	5808 (30.5)	4301 (29.7)	0.02
1	2718 (8.1)	1528 (8.0)	1190 (8.2)	−0.01
≥ 2	20,741 (61.8)	11,729 (61.5)	9012 (62.1)	−0.01
Angus score on admission				
0–1	16,796 (50.0)	9509 (49.9)	7287 (50.2)	−0.01
2	13,727 (40.9)	7820 (41.0)	5907 (40.7)	0.01
3–6	3045 (9.1)	1736 (9.1)	1309 (9.0)	0
Barthel index				
Total and severe dependence (0–55)	12,539 (43.9)	7055 (43.5)	5484 (44.5)	−0.02
Moderate and slight dependence (60–95)	1907 (6.7)	1125 (6.9)	782 (6.3)	0.02
Independence (100)	14,101 (49.4)	8030 (49.5)	6071 (49.2)	0.01
Emergency admission	22,421 (66.8)	12,752 (66.9)	9669 (66.7)	0
Ambulance use	16,286 (48.5)	9240 (48.5)	7046 (48.6)	0
Previous location before admission				
Home	29,609 (88.2)	16,816 (88.2)	12,793 (88.2)	0
Home based care service use before hospital admission	1113 (3.3)	664 (3.5)	449 (3.1)	0.02
Days from hospitalization to ICU admission, mean (SD)	5.05 (10.43)	5.08 (10.61)	5.01 (10.18)	0.01
Drugs within 2 days of admission				
Vasopressors	19,966 (59.5)	11,389 (59.7)	8577 (59.1)	0.01
Corticosteroids	11,155 (33.2)	6200 (32.5)	4955 (34.2)	−0.04
Neuromuscular blocking agents	19,845 (59.1)	11,272 (59.1)	8573 (59.1)	0
Aminoglycosides	1531 (4.6)	850 (4.5)	681 (4.7)	−0.01
Procedures within 2 days of admission				
Mechanical circulatory support	3079 (9.2)	1759 (9.2)	1320 (9.1)	0
Renal replacement therapy	1477 (4.4)	833 (4.4)	644 (4.4)	0
Mechanical ventilation	20,193 (60.2)	11,499 (60.3)	8694 (59.9)	0.01
Nasal intermittent positive pressure ventilation	194 (0.6)	107 (0.6)	87 (0.6)	0
High-flow nasal cannula	1357 (4.0)	691 (3.6)	666 (4.6)	−0.05
Blood transfusion	14,688 (43.8)	8356 (43.8)	6332 (43.7)	0
Primary diagnosis (ICD-10)				
Circulatory system	22,454 (66.9)	12,824 (67.3)	9630 (66.4)	0.03
Digestive system	1212 (3.6)	673 (3.5)	539 (3.7)	−0.01
Infectious and parasitic disease	506 (1.5)	293 (1.5)	213 (1.5)	0
Neoplasms	4496 (13.4)	2607 (13.7)	1889 (13.0)	0.03
Nervous system	329 (1.0)	172 (0.9)	157 (1.1)	−0.03
Respiratory system	1547 (4.6)	880 (4.6)	667 (4.6)	0
Other codes	3024 (9.0)	1616 (8.5)	1408 (9.7)	−0.06
Hospital volume, mean (SD)	258.61 (155.59)	265.57 (142.60)	249.46 (170.75)	0.1
Hospital type (academic)	12,305 (36.7)	6815 (35.7)	5490 (37.9)	−0.05
Patient-to-nurse ratio, mean (SD)	1.12 (0.40)	1.11 (0.34)	1.13 (0.46)	−0.02
Intensivist certified hospital	20,882 (62.2)	11,762 (61.7)	9120 (62.9)	−0.05
Dedicated therapist	4288 (12.8)	2166 (11.4)	2122 (14.6)	−0.17

### Changes in clinical interventions

We found that the proportion of patients who received some mobilization and rehabilitation interventions within 48 h of ICU admission increased from 41.3 to 73.3% in the pre- and post-introduction period. The introduction of financial incentives yielded EMR implementation in more patients admitted in the ICU.

### Changes in clinical outcomes

**Fig. 2:**
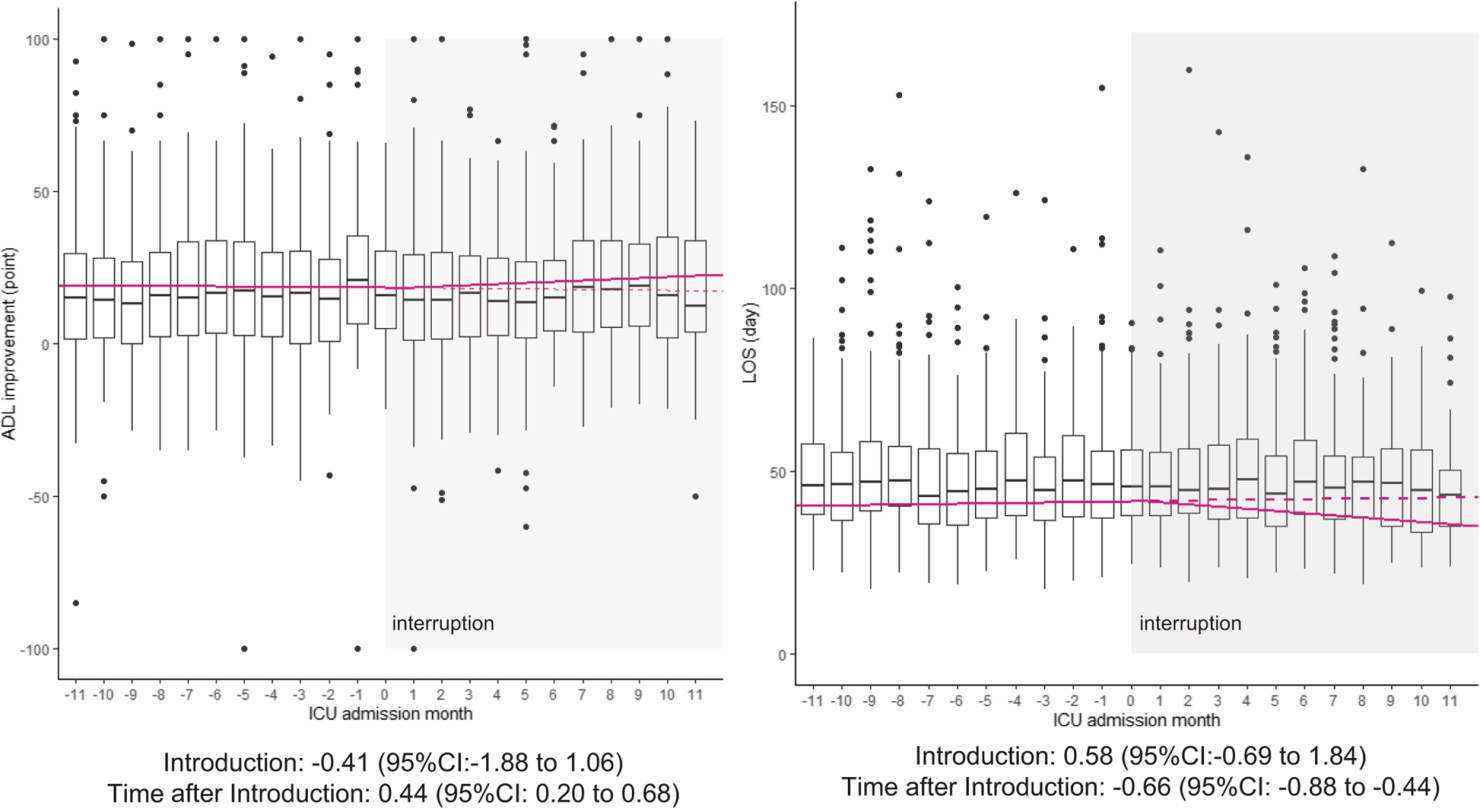
Changes in ADL improvement at discharge and LOS of hospital admission. Interrupted time-series of ADL improvement at hospital discharge and LOS during the pre- and post-introduction periods of the financial incentive. X-axis indicates the ICU admission month, which signifies the elapsed time from the introduction of the financial incentives for each hospital. Y-axis indicates the mean adjusted outcomes in each month. Red lines depict the interrupted time-series regression model describing outcomes before and after the introduction of the incentive. Dashed red lines depict the counterfactual outcomes in the absence of the introduction of the incentive. The box plots show the mean of each outcome by hospital per month. Boxes indicate first quartile, median, and third quartile. Dots denote observations outside the range of 1.5 × interquartile range [IQR]. A light shade of time periods visualizes the time after interruption; the introduction of incentive per each hospital. The outcomes were adjusted for age, sex, body mass index, smoking status, Charlson comorbidity index, Angus organ score, Barthel index at admission, emergency admission, ambulance use, numbers of days hospitalization until ICU admission, drugs and procedures within 2 days of hospital admission, hospital volume, patient-to-nurse ratio, intensivist certified hospital, and dedicated therapist. The main finding is that the financial incentive was associated with a significant slope change in improvement in the Barthel index at discharge and shorter length of stay at the hospital

We confirmed that the basic assumption of ITS was fulfilled. Seasonality was assessed through outcome plots per month (see additional file: Fig. S3). Additionally, we assumed no auto-correlation due to the independent nature of data. Figure 2 shows the interrupted time-series plots of changes in the outcomes (the BI at discharge and hospital LOS). The introduction of the financial incentive scheme was associated with improved BI at discharge (slope changes per month: 0.44 points; 95% CI = 0.20–0.68), and shorter hospital LOS (slope changes per month: − 0.66 days; 95% CI = − 0.88 to − 0.44 days). In contrast, there were no significant level changes in BI at discharge (− 0.41 points; 95% CI = − 1.88-1.06), and hospital LOS (0.58 days; 95% CI = − 0.69-1.84).

### Sensitivity analysis

Compared to before and after the introduction of the incentive, which did not assume a time series, there was a significant reduction in LOS (− 1.44 days, 95% CI: − 2.21, − 0.68 days). In contrast, ADLs at discharge increased similarly to the main analysis, but this was not significant (0.79 points, 95% CI: − 0.08, 1.67 points). The results of the interrupted time-series analyses between the pre- and post-policy issue period showed significant downward level and upward slope changes for the BI at discharge, and significant upward level and downward slope changes for hospital LOS (see additional file: Tables S2, 3 and Fig. S4). These findings remained mostly consistent using several intervals after the introduction or policy issue of this financial incentive (see additional file: Table S4). The combined datasets from 0 to 12 months for the prior period and 0–6 months for the posterior period showed exceptionally similar, although not significant, trends (see additional file: Fig. S5). Moreover, the sensitivity analysis using multiple imputations showed similar results to the main analysis model (see additional file: Fig. S6). In addition, the hospital volume demonstrated no clear trend between the baseline outcomes, thus the differences in baseline outcome values among facilities were negligible (see additional file: Fig. S7). Similarly, there was no clear trend between the proportion of patients claiming the incentive per hospital and the outcomes (see additional file: Fig. S8).

### Subgroup analysis

The results of the subgroup analyses stratified by age, type of admission, BI at admission, Angus organ failure score, and primary diagnosis are shown in the additional file: Table S4. The strongest significant effects in the slope change of BI at discharge and the hospital LOS was in patients who required total assistance at admission, and younger than 75 years old respectively. Compared to the main analysis, no subgroups showed a larger effect consistently.

## Discussion

The introduction of the financial incentive scheme was associated with improved BI at discharge and shorter hospital LOS. This financial incentive scheme for EMR in Japan was unique in that the target population includes all ICU patients, rather than those restricted by disease name or operative procedures. Additionally, not limiting rehabilitation providers to therapists may have increased opportunities for EMR intervention. Sensitivity analyses confirmed the robustness of these findings. The calculated annualized slope changes for BI, nearly 5 points upward, implied a clinically meaningful difference. Nevertheless, in the light of the absence of consistently stronger effects in any subgroup analysis compared to the main analysis, we inferred that this incentive system may have a broad impact, contributing to the benefit of the entire ICU patient population, rather than being limited to a specific patient subgroup. Thereby, the effect of the incentive payment policy may appear small at the population level.

Our findings have several explanations. First, some hospitals did not eventually claim the financial incentive fee possibly because of the suboptimal incentive design of insufficient monetary value. Only 62% of the patients admitted to hospitals with the financial scheme during the post-introduction periods claimed this financial scheme. Currently, two distinct categories of rehabilitation fees currently exist: per diem and fee-for-service payment structures, presenting a potential disparity. The financial incentive for EMR was designed as per diem payment scheme and could not be claimed on the same day as the existing rehabilitation fee. Consequently, this incentive might have resulted in lower overall payments to hospitals and may not have been effective in encouraging healthcare providers to enhance the quality of care. Coordination with other rehabilitation fees may be required to enhance this financial incentive.

Second, some hospitals may have not introduced the financial incentive scheme because they did not meet the facility-based criteria requirements. Only 25% of the patients within the post-issue periods were provided with this scheme. The difficulty in introducing the scheme may be attributed to a shortage of therapists and intensivists. A previous report showed that hospitals without this financial incentive scheme reported “the lack of therapists and intensivists” as the reason [48]. The present study indicated that the hospitals without this financial incentive scheme tended to be lacking certified intensivists (see additional file: Table S5). Expanding the job assignments of certified intensivists may be necessary to increase the number of EMR intervention hospitals.

To the best of our knowledge, this is the first study to examine the effect of the financial incentive scheme for EMR on patient outcomes. The present study has important clinical and policy implications using interrupted time-series analyses with national data. The implementation of EMR has many barriers despite its benefits and safety [11], and the financial incentive could potentially be the solution. Our study suggests that the introduction of a financial incentive scheme for providing EMR in the ICU may be associated with improved ADL at discharge and shortened hospital LOS.

### Potential limitations

Our study has some limitations. First, the Barthel Index may lack sensitivity as an effect measure due to ceiling effects. Nevertheless, alternative indices such as BI Effectiveness and BI Efficiency [43] are uncommon in the intensive care field. The current outcome measure is unlikely to influence the interpretation of the findings significantly, as it introduces a bias that tends to underestimate the observed effect. Second, we adjusted the model using baseline patient characteristics to ensure the basic assumption of the ITS, which assume that the characteristics of the population remained unchanged throughout the study period. However, this is not well-established approach. Third, we only evaluated short-term outcomes within 1 year after the introduction of the financial incentive. The potential time required for substantial improvements in specialist team function and the refinement of protocols and interventions might extend beyond the study period. However, the post-study period following our current setting might not fulfill the basic assumptions of ITS due to the impact of the COVID-19 pandemic in the winter of 2019. Fourth, other co-occurring events may have affected patient outcomes at the time the financial incentive was introduced. Notably, these included the medical reimbursement revision in April 2018, and the “Medical Cost Optimization Plan” [49, 50]. In April 2018, the requirement criteria for “assignment of full-time advanced practice nurse” as ICU facility criterion by Ministry of Health, Labour and Welfare. However, the criteria provided hospitals with grace periods of 2 years after the policy was issued. Moreover, previous studies reported that the association between advanced practice nurse and shorter LOS were not consistent [51–53]. We attempted to remove the impact of this co-occurring events, by choice of a design that set the study population to patients admitted with the financial incentive for EMR, and the first day of each hospital’s incentive claim. Furthermore, the “Medical Cost Optimization Plan” designed to cut medical costs from fiscal year 2013 to 2017, included a target of reducing hospital LOS. However, the LOS trend was assumed to be constant and ended in the middle of the study period. Therefore, we assumed that this co-occurring event may not have a significant impact on this study, especially in effect to the primary outcome. Fifth, we lacked detailed EMR information, such as frequency, intensity, time, and type. There is currently no structure in place to guarantee the program or quality of the EMR. Hence, we were unable to evaluate the concrete intervention changes affected by the financial incentive scheme. The potential unmeasured time-varying confounders, such as recent alterations in routine care might have impacted the study. Therefore, caution is warranted in interpreting the clinical outcomes. Last, our study was set around the Japanese universal healthcare coverage system and the results may not be generalizable to other situations. In particular, the average LOS in acute care hospitals in Japan is much longer than in other countries [54]. Thus, we used an analytical method and conducted sensitivity analysis to consider the impact of very long hospital stays.

## Conclusions

The study suggests a potential association between the financial incentive for EMR in ICU patients and improved outcomes by using nationally representative data. This incentive scheme may provide a unique solution to EMR barrier in practice, however, caution is warranted in interpreting these findings due to recent changes in ICU care practices.

### Supplementary Information

**Supplementary Material 1.**

## Data Availability

The datasets generated and /or analysed during the current study are not publicly available due to contracts with the hospitals providing data for the database.

## Abbreviations

ADL: Activities of daily living
BI: Barthel index
CI: Confidence interval
DPC: Diagnosis procedure combination
EMR: Early mobilization and rehabilitation
ICU: Intensive care unit
ICD-10: International classification of diseases 10th revision
JIPAD: Japanese intensive care patient database
LOS: Length of stay
USD: United States dollar.
