# Supplementary material for: Exploring the influence of a financial incentive scheme on early mobilization and rehabilitation in ICU patients: an interrupted time-series analysis
Source: BMC Health Serv Res. 2024 Feb 24;24:242. doi: 10.1186/s12913-024-10763-0 (PMC10893682; doi:10.1186/s12913-024-10763-0)
Supplement: Supplementary file 1 — Supplementary Material 1. [file 12913_2024_10763_MOESM1_ESM.docx]

***Additional File: Figures and Tables***

**Exploring the influence of a financial incentive scheme on early mobilization and rehabilitation in ICU patients: An interrupted time-series analysis**

Yoko Unoki, MPH ^1^, Sachiko Ono, MPH, PhD^2,*^, Yusuke Sasabuchi, MD, MPH, PhD ^3,*^, Yohei Hashimoto, MD, PhD ^4,5^, Hideo Yasunaga, MD, PhD ^5^, Isao Yokota, MPH, PhD ^1^

* Equally contributed

1. Department of Biostatistics, Graduate School of Medicine, Hokkaido University, N15W7, Kita-ku, Sapporo, Hokkaido, Japan, 0608638

Email: yokotai@pop.med.hokudai.ac.jp

1. Department of Eat-loss Medicine, Graduate School of Medicine, The University of Tokyo, Tokyo, Japan.
2. Department of Real-world Evidence, Graduate School of Medicine, The University of Tokyo, Tokyo, Japan.
3. Department of Ophthalmology, Graduate School of Medicine, The University of Tokyo, Tokyo, Japan.
4. Department of Clinical Epidemiology and Health Economics, School of Public Health, The University of Tokyo, Tokyo, Japan.

**List of Contents**

**Supplementary the statistical analysis section**

**Supplemental Figures:**

**Fig S1.** Causal diagrams

**Fig S2.** Sensitivity analysis with several time intervals

**Fig S3.** Changes in ADL improvement at discharge and LOS of hospital admission

**Fig S4.** Flowchart of patients in the sensitivity analysis

**Fig S5.** Changes in ADL improvement at discharge and hospital LOS in sensitivity analysis

**Fig S6.** Changes in ADL improvement at discharge and hospital LOS using multiple imputations

**Fig S7.** Scatter plot of Hospital volume vs. Outcomes

**Fig S8.** Scatter plot of Proportion of patients claimed EMR incentive vs. Outcomes

**Supplemental Tables:**

**Table S1.** ICD-10 code, Japanese procedure codes, and drugs used to define the Angus organ failure score

**Table S2.** Patient characteristics in the sensitivity analysis

**Table S3.** Level changes and slope changes in outcomes between the pre-and post-introduction, and the pre-and post-policy issue periods on several intervals

**Table S4.** Level changes and slope changes in outcomes between the pre-and post-introduction periods for subgroup analyses

**Table S5.** Patient characteristics in the sensitivity analyses

**SUPPLEMENTARY THE STATISTICAL ANALYSIS SECTION**

**Sensitivity analyses**

***Compared to before and after the incentive without time-series***

We compared to before and after the introduction of the incentive, which did not assume a time series.

***ITS analyses changed the study population and the time of origin***

First, we conducted the same analysis as the main analysis with the entire study population, including patients admitted to hospitals that either introduced or not the incentive within the study period. We set the issue of the financial incentive scheme on April 1, 2018, as the time origin.

***ITS analyses at several intervals***

We conducted the analyses at several intervals, and used the date of the introduction and the policy issue date of the financial incentive scheme as the time origin. We calculated the months of ICU admissions, which represented the time elapsed since time origin. We performed five analyses, each combining data with pre-periods (0–12 months, 3–12 months, and 6–12 months) and post-periods (0−6months, 3–12 months, and 6–12 months) (see additional file: **Fig. S2**).

***Multiple imputations***

We fitted the mixed effect model using multiple imputations by chained equations. Regarding LOS, we analyzed the entire group, comprising patients with very long hospitalizations (≥ 90 days) and patients who were hospitalized for < 90 days.

***Correlations between variables***

We determined whether there is a correlation between outcome and hospital volume. We also plotted outcomes versus the proportion of patients having claimed the incentives per hospital to determine any correlation.

**Fig. S1.** Causal diagrams

Abbreviations: ICUAW, ICU acquired weakness; EMR, early mobilization/ rehabilitation; MOF, multiple organ failure

**Fig. S2.** Sensitivity analysis with several time intervals

Sensitivity analysis combining data with pre-periods (0–12 months, 3–12 months, and 6–12 months) and post-periods (0-6months, 3–12 months, and 6–12 months). The red vertical line indicates the time origin. For pre-and post-periods, we represented the time elapsed in month since the introduction or April 2018 when the financial incentive policy was issued.

**Fig. S3** Changes in ADL improvement at discharge and LOS of hospital admission.

Interrupted time-series of ADL improvement at hospital discharge and LOS during the pre- and post-introduction periods of the financial incentive. The dots and error bars indicate the mean outcomes in each month and standard deviations. The vertical dashed line indicates the introduction of financial incentives for each hospital. Regression models are fitted to the plots, and the grey areas show 95% confidence intervals. The X-axis indicates the ICU admission month, which indicates the elapsed time from the introduction of the financial incentives for each hospital. The outcomes were adjusted for age, sex, body mass index, smoking status, Charlson comorbidity index, Angus organ score, Barthel index at admission, emergency admission, ambulance use, numbers of days hospitalization until ICU admission, drugs and procedures within two days of hospital admission, hospital volume, patient-to-nurse ratio, intensivist certified hospital, and dedicated therapist.

**Fig. S4.** Flowchart of patients in the sensitivity analysis


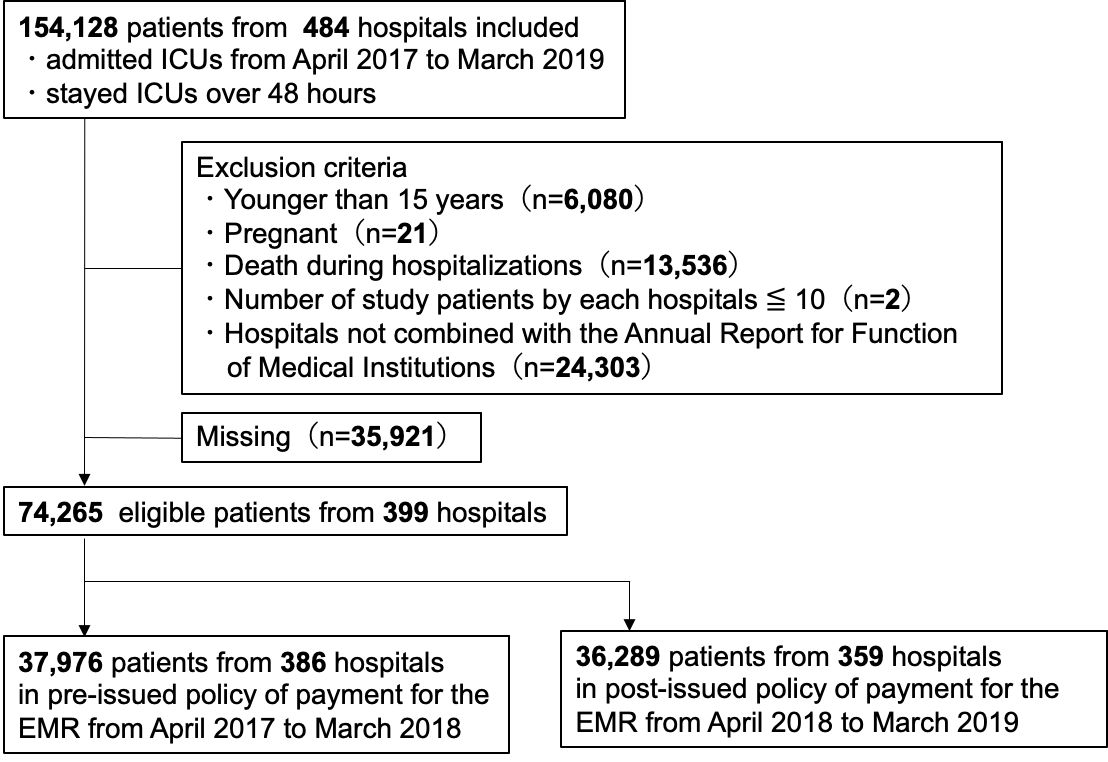

**Fig. S5.** Changes in ADL improvement at discharge and hospital LOS in sensitivity analysis

Abbreviations: ADL, activities of daily living; LOS, length of stay; SA, sensitivity analysis

Interrupted time-series of ADL improvement at hospital discharge and LOS during the pre- and post-introduction periods of the financial incentive. X-axis indicates the ICU admission month, which indicates the elapsed time from the introduction of the financial incentives for each hospital. Y-axis indicates the mean adjusted outcomes in each month. Box plots showing the mean of each outcome by hospital per month. Boxes indicate first quartile, median, and third quartile. Dots denote observations outside the range of 1.5 × interquartile range. A light shade of time periods visualizes the time after interruption; the introduction of incentive per each hospital. Red lines depict the the interrupted time series regression model describing outcomes. Red dashed lines depict the mixed effect model using combining data with whole pre-periods (0–12 months) and post-periods (0-6 months). The outcomes were adjusted for age, sex, body mass index, smoking status, Charlson comorbidity index, Angus organ score, Barthel index at admission, emergency admission, ambulance use, numbers of days hospitalization until ICU admission, drugs and procedures within two days of hospital admission, hospital volume, patient-to-nurse ratio, intensivist certified hospital, and dedicated therapist.

**Fig. S6.** Changes in ADL improvement at discharge and hospital LOS using multiple imputations


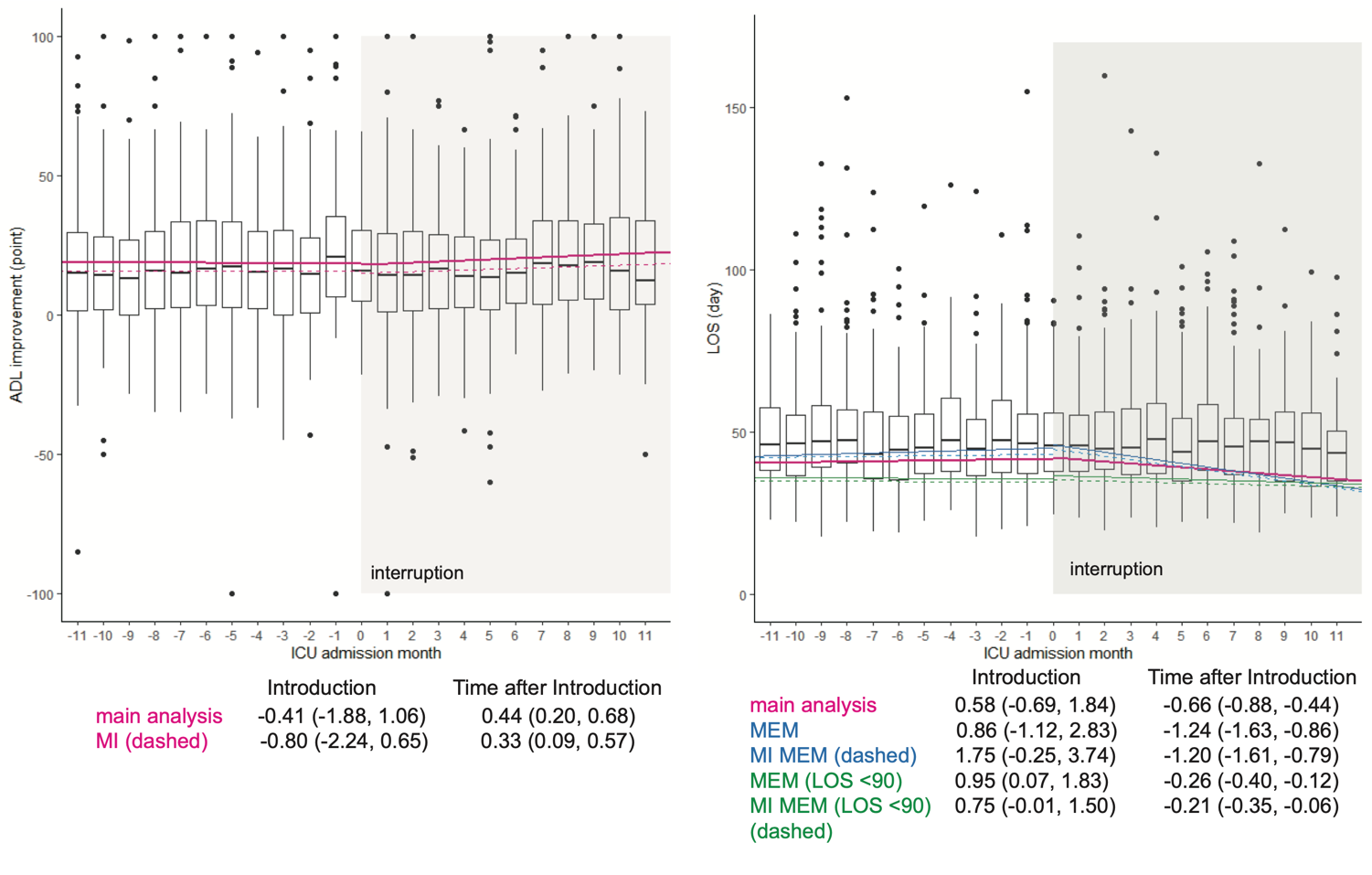


Abbreviations: ADL, activities of daily living; MI, multiple imputations; MEM, multilevel effect model; LOS, length of stay

Interrupted time-series of ADL improvement at hospital discharge and LOS during the pre- and post-introduction periods of the financial incentive. X-axis indicates the ICU admission month, which indicates the elapsed time from the introduction of the financial incentives for each hospital. Y-axis indicates the mean adjusted outcomes in each month. Box plots showing the mean of each outcome by hospital per month. Boxes indicate first quartile, median, and third quartile. Dots denote observations outside the range of 1.5 × interquartile range. A light shade of time periods visualizes the time after interruption; the introduction of incentive per each hospital. Red lines depict the interrupted time series regression model describing outcomes before and after the introduction of the incentive. Regarding LOS, the green line depicts the mixed-effect model for all patients, and the blue line depicts the mixed-effect model for patients whose LOS was under 90 days. All dashed lines depict the mixed effect model using the multiple imputations by chained equations. The outcomes were adjusted for age, sex, body mass index, smoking status, Charlson comorbidity index, Angus organ score, Barthel index at admission, emergency admission, ambulance use, numbers of days hospitalization until ICU admission, drugs and procedures within two days of hospital admission, hospital volume, patient-to-nurse ratio, intensivist certified hospital, and dedicated therapist.

**Fig. S7.** Scatter plot of Hospital volume vs. Outcomes

Abbreviations: ADL, activities of daily living; LOS, length of stay

Scatter plot of the baseline outcomes and hospital volume. The X-axis indicates hospital volume which was calculated as the sum of the number of study patients per hospital during the pre-introduction periods. The Y-axis indicates the mean outcomes by hospitals during the pre-introduction periods.

**Fig. S8.** Scatter plot of Days between the policy issue date and the first date claimed incentives for each hospital vs. Outcomes

Abbreviations: ADL, activities of daily living; LOS, length of stay; EMR, early mobilization/ rehabilitation

Scatter plot of the baseline outcomes and days between the policy issue date and the first date claimed incentives for each hospital. The X-axis indicates the proportion of patients having claimed EMR incentives per hospital during the post-introduction periods. The Y-axis indicates the mean outcomes by hospitals during the pre-introduction periods.

**Table S1.** ICD-10 code, Japanese procedure codes, and drugs used to define the Angus organ failure score

| Organ failure | ICD-10 codes | Japanese procedure codes or claim |
| --- | --- | --- |
| Cardiovascular | I95, R57 | Vasopressor or inotrope (dopamine, epinephrine, norepinephrine, or vasopressin), |
|  |  | K602 (extracorporeal membrane oxygenation) |
| Respiratory |  | J045 (mechanical ventilation) |
| Neurologic | F05, G93.1, G93.4 |  |
| Hematologic | D65, D68.8, D68.9, D69.5, D69.6 |  |
| Hepatic | K72.0, K76.2, K76.3 |  |
| Renal | N17 | J038 (renal replacement therapy) |

Abbreviations; ICD-10, International Classification of Diseases, 10th revision

**Table S2.** Patient characteristics in the sensitivity analysis

|  | Overall | Pre-issue | Post-issue |  |
| --- | --- | --- | --- | --- |
|  |  | period | period |  |
|  | (399 hospitals) | (386 hospitals) | (359 hospitals) | Std |
| Patient characteristics | (n=74,265) | (n=37,976) | (n=36,289) | diff |
| Age, (mean (SD)) | 70.55 (13.41) | 70.52 (13.35) | 70.59 (13.46) | -0.01 |
| Male sex | 46624 (62.8) | 23945 (63.1) | 22679 (62.5) | 0.01 |
| Body mass index (kg/㎡), (mean (SD)) | 22.84 (5.27) | 22.79 (4.76) | 22.89 (5.75) | -0.02 |
| Smoking Status |  |  |  |  |
| Current / Past smoker | 38632 (52.0) | 19767 (52.1) | 18865 (52.0) | 0.00 |
| Charlson Comorbidity Index |  |  |  |  |
| 0 | 23171 (31.2) | 12066 (31.8) | 11105 (30.6) | 0.03 |
| 1 | 5967 (8.0) | 2968 (7.8) | 2999 (8.3) | -0.02 |
| ≥2 | 45127 (60.8) | 22942 (60.4) | 22185 (61.1) | -0.01 |
| Angus score on admission |  |  |  |  |
| 0-1 | 37545 (50.6) | 19461 (51.2) | 18084 (49.8) | 0.03 |
| 2 | 29975 (40.4) | 15186 (40.0) | 14789 (40.8) | -0.01 |
| 3-6 | 6745 (9.1) | 3329 (8.8) | 3416 (9.4) | -0.03 |
| Barthel index |  |  |  |  |
| Total and severe dependence  (0-55) | 28765 (44.7) | 14697 (44.7) | 14068 (44.8) | 0.00 |
| Moderate and slight dependence  (60-95) | 4511 (7.0) | 2297 (7.0) | 2214 (7.1) | 0.00 |
| Independence (100) | 31027 (48.3) | 15921 (48.4) | 15106 (48.1) | 0.01 |
| Emergency admission | 48801 (65.7) | 25095 (66.1) | 23706 (65.3) | 0.02 |
| Ambulance use | 35131 (47.3) | 17893 (47.1) | 17238 (47.5) | -0.01 |
| Previous location before admission |  |  |  |  |
| Home | 65562 (88.3) | 33592 (88.5) | 31970 (88.1) | 0.01 |
| Home based care service use before hospital admission | 2409 (3.2) | 1238 (3.3) | 1171 (3.2) | 0.01 |
| Days of hospitalization until ICU admission, (mean (SD)) | 5.22 (11.13) | 5.25 (10.46) | 5.18 (11.80) | 0.01 |
| Drugs within 2 days hospital admission |  |  |  |  |
| Vasopressors | 44366 (59.7) | 22440 (59.1) | 21926 (60.4) | -0.03 |
| Corticosteroids | 24307 (32.7) | 11977 (31.5) | 12330 (34.0) | -0.05 |
| Neuromuscular blocking agents | 44908 (60.5) | 22698 (59.8) | 22210 (61.2) | -0.03 |
| Aminoglycosides | 3366 (4.5) | 1798 (4.7) | 1568 (4.3) | 0.02 |
| Procedures within 2 days hospital admission | |  |  |  |
| Mechanical circulatory support | 6607 (8.9) | 3420 (9.0) | 3187 (8.8) | 0.01 |
| Renal replacement therapy | 3270 (4.4) | 1628 (4.3) | 1642 (4.5) | -0.01 |
| Mechanical ventilation | 43877 (59.1) | 22175 (58.4) | 21702 (59.8) | -0.03 |
| NIPPV | 453 (0.6) | 232 (0.6) | 221 (0.6) | 0.00 |
| HFNC | 2798 (3.8) | 1287 (3.4) | 1511 (4.2) | -0.04 |
| Blood transfusion | 32511 (43.8) | 16370 (43.1) | 16141 (44.5) | -0.03 |
| Primary diagnosis (ICD-10) |  |  |  |  |
| Circulatory system | 48802 (65.7) | 25185 (66.3) | 23617 (65.1) | 0.04 |
| Digestive system | 2730 (3.7) | 1346 (3.5) | 1384 (3.8) | -0.02 |
| Infectious and parasitic disease | 1079 (1.5) | 550 (1.4) | 529 (1.5) | -0.01 |
| Neoplasms | 10823 (14.6) | 5543 (14.6) | 5280 (14.5) | 0.00 |
| Nervous system | 750 (1.0) | 356 (0.9) | 394 (1.1) | -0.03 |
| Respiratory system | 3326 (4.5) | 1733 (4.6) | 1593 (4.4) | 0.01 |
| Other codes | 6755 (9.1) | 3263 (8.6) | 3492 (9.6) | -0.05 |
| Hospital volume, (mean (SD)) | 244.16 (165.61) | 242.50 (156.74) | 245.90 (174.39) | -0.02 |
| Hospital type (academic) | 24383 (32.8) | 11877 (31.3) | 12506 (34.5) | -0.07 |
| Patient-to-nurse ratio, (mean (SD)) | 1.16 (0.46) | 1.15 (0.42) | 1.18 (0.49) | -0.05 |
| Intensivist certified hospital | 41701 (56.2) | 20833 (54.9) | 20868 (57.5) | -0.07 |
| Dedicated therapist | 7012 (9.4) | 2818 (7.4) | 4194 (11.6) | -0.13 |

Data were presented as unweighted number (percentage) of patients unless otherwise indicated.

Abbreviations: Std diff, standardized difference; SD, standard deviation; ICU, intensive care unit; NIPPV, nasal intermittent positive pressure ventilation; HFNC, high-flow nasal cannula

**Table S3.** Level changes and slope changes in outcomes between the pre-and post-introduction, and the pre-and post-policy issue periods in several intervals

|  | Level change (95%CI) | | |  |  | Slope change (95%CI) | | |  |
| --- | --- | --- | --- | --- | --- | --- | --- | --- | --- |
| Outcomes / study periods | incentive introduction | | policy issue | |  | incentive introduction | | policy issue | |
| ADL improvement (point) |  |  |  |  |  |  |  |  |  |
| main analysis | -0.41 | ( -1.88 to 1.06) | -2.32 | ( -3.34 to -1.29) |  | 0.44 | (0.20 to 0.68) | 0.18 | (0.03 to 0.33) |
| pre0-12/ post3-12 | -0.07 | ( -1.73 to 1.59) | -2.79 | ( -3.91 to -1.67) |  | 0.59 | (0.24 to 0.93) | 0.45 | (0.25 to 0.65) |
| pre3-12/ post3-12 | 0.00 | ( -1.76 to 1.77) | -3.27 | ( -4.47 to -2.08) |  | 0.71 | (0.32 to 1.10) | 0.35 | (0.11 to 0.58) |
| pre0-12/ post6-12 | 0.16 | ( -1.85 to 2.18) | -2.56 | ( -3.81 to -1.30) |  | 1.03 | (0.39 to 1.68) | 0.85 | (0.51 to 1.19) |
| pre6-12/ post6-12 | 0.29 | ( -2.00 to 2.58) | -2.89 | ( -4.34 to -1.44) |  | 1.23 | (0.46 to 1.99) | 0.86 | (0.42 to 1.29) |
| pre0-12/ post0-6 | 0.60 | (-1.18 to 2.39) | -0.83 | (-2.05 to 0.39) |  | 0.08 | (-0.37 to 0.53) | -0.26 | (-0.61 to 0.09) |
| Length of hospital stay (day) |  |  |  |  |  |  |  |  |  |
| main analysis | 0.44 | ( -0.69 to 1.84) | 2.44 | (1.51 to 3.38) |  | -0.66 | ( -0.88 to -0.44) | -0.54 | ( -0.68 to -0.40) |
| pre0-12/ post3-12 | 0.59 | ( -0.31 to 2.54) | 2.99 | (1.98 to 4.00) |  | -1.13 | ( -1.47 to -0.80) | -0.96 | ( -1.17 to -0.75) |
| pre3-12/ post3-12 | 0.71 | ( -0.51 to 2.48) | 3.03 | (1.99 to 4.08) |  | -1.26 | ( -1.63 to -0.89) | -1.01 | ( -1.24 to -0.78) |
| pre0-12/ post6-12 | 1.03 | ( -0.41 to 2.87) | 3.44 | (2.42 to 4.45) |  | -2.15 | ( -2.70 to -1.59) | -1.94 | ( -2.27 to -1.61) |
| pre6-12/ post6-12 | 1.23 | ( -0.94 to 2.67) | 3.37 | (2.25 to 4.49) |  | -2.62 | ( -3.31 to -1.94) | -2.21 | ( -2.61 to -1.80) |
| pre0-12/ post0-6 | -0.92 | (-2.31 to 0.47) | -0.36 | (-1.40 to 0.68) |  | -0.04 | (-0.41 to 0.33) | 0.11 | (-0.13 to 0.35) |

Abbreviations: CI, confidence interval

The outcomes were adjusted for age, sex, BMI, smoking status, Charlson comorbidity index, Angus organ score, Barthel index at admission, emergency admission, ambulance, numbers of days hospitalization until ICU admission, drugs, and procedures within 2 days of hospital admission (vasopressors, corticosteroids, neuromuscular blocking agents, aminoglycosides, mechanical circulatory support, renal replacement therapy, mechanical ventilation, and blood transfusion), hospital volume, patient-to-nurse ratio, intensivist certified hospital, and dedicated therapist.

**Table S4.** Level changes and slope changes in outcomes between the pre-and pot- introduction periods for subgroup analysis

|  |  | | ADL improvement（point） | | | |  |  | Length of hospital stay（day） | | | | |
| --- | --- | --- | --- | --- | --- | --- | --- | --- | --- | --- | --- | --- | --- |
| Subgroup |  | | Level change (95%CI) | |  | Slope change (95%CI) | |  | Level change (95%CI) | |  | Slope change (95%CI) | |
| Main analysis |  | | -0.41 | (-1.88 to 1.06) |  | 0.44 | (0.20 to 0.68) |  | 0.58 | (-0.69 to 1.84) |  | -0.66 | (-0.88 to -0.44) |
| Age | ≥75 | 0.27 | | (-2.03 to 2.56) |  | 0.45 | (0.08 to 0.82) |  | 0.95 | (-0.71 to 2.62) |  | -0.38 | (-0.65 to -0.11) |
|  | <75 | | -1.01 | (-2.87 to 0.84) |  | 0.38 | (0.08 to 0.69) |  | 0.17 | (-1.44 to 1.78) |  | -0.76 | (-1.02 to -0.50) |
| Emergency admission | Yes | | -0.33 | (-2.43 to 1.78) |  | 0.34 | (0.00 to 0.68) |  | -0.14 | (-1.65 to 1.37) |  | -0.61 | (-0.85 to -0.37) |
|  | No | | -0.37 | (-1.96 to 1.21) |  | 0.26 | (-0.02 to 0.53) |  | 1.64 | (0.07 to 3.22) |  | -0.54 | (-0.83 to -0.24) |
| Barthel index | Total assistance | | -1.13 | (-3.91 to 1.65) |  | 0.66 | (0.22 to 1.11) |  | 0.63 | (-1.07 to 2.34) |  | -0.60 | (-0.89 to -0.31) |
|  | Partial assistance | | -0.10 | (-5.62 to 5.41) |  | -0.31 | (-1.24 to 0.61) |  | -1.69 | (-6.09 to 2.71) |  | -0.75 | (-1.44 to -0.05) |
|  | Independent | | 0.51 | (-0.86 to 1.88) |  | 0.20 | (-0.03 to 0.42) |  | 0.41 | (-1.10 to 1.91) |  | -0.46 | (-0.70 to -0.23) |
| Angus organ failure score | ≥3 | | 0.65 | (-4.45 to 5.75) |  | 0.65 | (-4.45 to 5.75) |  | -2.11 | (-5.78 to 1.57) |  | -0.64 | (-1.23 to -0.05) |
|  | <3 | | -0.45 | (-1.98 to 1.08) |  | 0.48 | (0.23 to 0.73) |  | 0.79 | (-0.50 to 2.08) |  | -0.62 | (-0.84 to -0.41) |
| Primary diagnosis | Circulatory system | | -0.36 | (-2.14 to 1.42) |  | 0.28 | (-0.01 to 0.57) |  | 1.11 | (-0.19 to 2.41) |  | -0.43 | (-0.63 to -0.23) |
|  | Digestive system | | 1.25 | (-6.93 to 9.44) |  | 0.43 | (-0.92 to 1.78) |  | 1.96 | (-3.90 to 7.83) |  | -0.25 | (-1.23 to 0.73) |
|  | Infectious diseases | | -3.99 | (-19.00 to 11.02) |  | 0.19 | (-2.32 to 2.69) |  | -2.20 | (-12.13 to 7.73) |  | 1.22 | (-0.43 to 2.88) |
|  | Nervous system | | 11.51 | (-7.04 to 30.06) |  | 1.07 | (-2.11 to 4.26) |  | 11.07 | (-4.34 to 26.47) |  | -1.74 | (-4.31 to 0.82) |
|  | Neoplasms | | 0.45 | (-2.58 to 3.47) |  | 0.37 | (-0.13 to 0.88) |  | -1.41 | (-1.41 to 1.68) |  | -0.67 | (-1.26 to -0.07) |
|  | Respiratory system | | 7.36 | (-1.16 to 15.87) |  | 0.81 | (-0.54 to 2.16) |  | -5.54 | (-10.84 to -0.24) |  | -0.48 | (-1.33 to 0.36) |
|  | Other diseases | | -1.41 | (-6.31 to 3.49) |  | 0.63 | (-0.17 to 1.43) |  | -3.54 | (-7.76 to 0.68) |  | -1.51 | (-2.15 to -0.88) |

Abbreviations: CI, confidence interval

The outcomes were adjusted for age, sex, BMI, smoking status, Charlson comorbidity index, Angus organ score, Barthel index at admission, emergency admission, ambulance, numbers of days hospitalization until ICU admission, drugs, and procedures within 2 days of hospital admission (vasopressors, corticosteroids, neuromuscular blocking agents, aminoglycosides, mechanical circulatory support, renal replacement therapy, mechanical ventilation, and blood transfusion), hospital volume, patient-to-nurse ratio, intensivist certified hospital, and dedicated therapist.

**Table S5.** Patient characteristics in the sensitivity analysis

|  | | Overall | Without financial | With financial |  |
| --- | --- | --- | --- | --- | --- |
|  | |  | incentive hospitals | incentive hospitals |  |
|  | | (399 hospitals) | (168 hospitals) | (205 hospitals) | Std |
| Patient characteristics | | (n=74,265) | (n=36,337) | (n=37,928) | diff |
| Age, (mean (SD)) | | 70.55 (13.41) | 70.74 (13.22) | 70.38 (13.58) | 0.03 |
| Male sex | | 46624 (62.8) | 22644 (62.3) | 23980 (63.2) | -0.02 |
| Body mass index (kg/㎡),  (mean (SD)) | 22.84 (5.27) | 22.79 (4.78) | 22.89 (5.69) | -0.02 |  |
| Smoking Status | |  |  |  |  |
| Current / Past smoker | | 38632 (52.0) | 18331 (50.4) | 20301 (53.5) | -0.06 |
| Charlson Comorbidity Index | |  |  |  |  |
| 0 | | 23171 (31.2) | 11578 (31.9) | 11593 (30.6) | 0.03 |
| 1 | | 5967 (8.0) | 2885 (7.9) | 3082 (8.1) | -0.01 |
| ≥2 | | 45127 (60.8) | 21874 (60.2) | 23253 (61.3) | -0.02 |
| Angus score on admission | |  |  |  |  |
| 0-1 | | 37545 (50.6) | 18564 (51.1) | 18981 (50.0) | 0.02 |
| 2 | | 29975 (40.4) | 14478 (39.8) | 15497 (40.9) | -0.02 |
| 3-6 | | 6745 (9.1) | 3295 (9.1) | 3450 (9.1) | 0.00 |
| Barthel index | |  |  |  |  |
| Total and severe dependence  　(0-55) | | 28765 (44.7) | 14465 (45.3) | 14300 (44.2) | 0.02 |
| Moderate and slight dependence  　(60-95) | | 4511 (7.0) | 2361 (7.4) | 2150 (6.6) | 0.03 |
| Independence (100) | | 31027 (48.3) | 15108 (47.3) | 15919 (49.2) | -0.04 |
| Emergency admission | | 48801 (65.7) | 23503 (64.7) | 25298 (66.7) | -0.04 |
| Ambulance use | | 35131 (47.3) | 16765 (46.1) | 18366 (48.4) | -0.05 |
| Previous location before admission | |  |  |  |  |
| Home | | 65562 (88.3) | 32158 (88.5) | 33404 (88.1) | 0.01 |
| Home based care service use before hospital admission | | 2409 (3.2) | 1172 (3.2) | 1237 (3.3) | -0.01 |
| Days of hospitalization until ICU admission, (mean (SD)) | | 5.22 (11.13) | 5.39 (11.87) | 5.06 (10.37) | 0.03 |
| Drugs within 2 days hospital admission | | |  |  |  |
| Vasopressors | | 44366 (59.7) | 21839 (60.1) | 22527 (59.4) | 0.01 |
| Corticosteroids | | 24307 (32.7) | 11696 (32.2) | 12611 (33.2) | -0.02 |
| Neuromuscular blocking agents | | 44908 (60.5) | 22444 (61.8) | 22464 (59.2) | 0.05 |
| Aminoglycosides | | 3366 (4.5) | 1689 (4.6) | 1677 (4.4) | 0.01 |
| Procedures within 2 days hospital admission | | |  |  |  |
| Mechanical circulatory support | | 6607 (8.9) | 3073 (8.5) | 3534 (9.3) | -0.03 |
| Renal replacement therapy | | 3270 (4.4) | 1613 (4.4) | 1657 (4.4) | 0.00 |
| Mechanical ventilation | | 43877 (59.1) | 21064 (58.0) | 22813 (60.1) | -0.04 |
| NIPPV | | 453 (0.6) | 232 (0.6) | 221 (0.6) | 0.00 |
| HFNC | | 2798 (3.8) | 1324 (3.6) | 1474 (3.9) | -0.02 |
| Blood transfusion | | 32511 (43.8) | 15942 (43.9) | 16569 (43.7) | 0.00 |
| Primary diagnosis (ICD-10) | |  |  |  |  |
| Circulatory system | | 48802 (65.7) | 23398 (64.4) | 25404 (67.0) | -0.05 |
| Digestive system | | 2730 (3.7) | 1380 (3.8) | 1350 (3.6) | 0.01 |
| Infectious and parasitic disease | | 1079 (1.5) | 498 (1.4) | 581 (1.5) | -0.01 |
| Neoplasms | | 10823 (14.6) | 5746 (15.8) | 5077 (13.4) | 0.07 |
| Nervous system | | 750 (1.0) | 385 (1.1) | 365 (1.0) | 0.01 |
| Respiratory system | | 3326 (4.5) | 1575 (4.3) | 1751 (4.6) | -0.01 |
| Other codes | | 6755 (9.1) | 3355 (9.2) | 3400 (9.0) | 0.01 |
| Hospital volume, (mean (SD)) | | 244.16 (165.61) | 231.02 (177.80) | 256.76 (151.95) | -0.16 |
| Hospital type (academic) | | 24383 (32.8) | 10453 (28.8) | 13930 (36.7) | -0.17 |
| Patient-to-nurse ratio, (mean (SD)) | | 1.16 (0.46) | 1.20 (0.50) | 1.13 (0.42) | 0.15 |
| Intensivist certified hospital | | 41701 (56.2) | 18111 (49.8) | 23590 (62.2) | -0.25 |
| Dedicated therapist | | 7012 (9.4) | 2544 (7.0) | 4468 (11.8) | -0.17 |

Data were presented as unweighted number (percentage) of patients unless otherwise indicated.

Abbreviations: Std diff, standardized difference; SD, standard deviation; ICU, intensive care unit; NIPPV, nasal intermittent positive pressure ventilation; HFNC, high-flow nasal cannulas
